# A Simple and Sensitive Nanogold RRS/Abs Dimode Sensor for Trace As^3+^ Based on Aptamer Controlled Nitrogen Doped Carbon Dot Catalytic Amplification

**DOI:** 10.3390/molecules26195930

**Published:** 2021-09-30

**Authors:** Hongyan Bai, Haolin Wang, Fuzhang Bai, Aihui Liang, Zhiliang Jiang

**Affiliations:** 1 Key Laboratory of Ecology of Rare and Endangered Species and Environmental Protection (Guangxi Normal University), Ministry of Education, Guilin 541004, China; hongyanbaia@163.com (H.B.); whl18934829352@163.com (H.W.); baixiaobaia2021@163.com (F.B.); 2 Guangxi Key Laboratory of Environmental Pollution Control Theory and Technology, Guilin 541004, China

**Keywords:** CD_N_, AuNPs, catalytic amplification, aptamer, RRS/Abs dual-mode sensor, As^3+^

## Abstract

Using citric acid (CA) and ethylenediamine (EDA) as precursors, stable nitrogen-doped carbon dots (CD) nanosols were prepared by microwave procedure and characterized in detail. It was found that CD_N_s catalyze ethanol (Et)-HAuCl_4_ to generate gold nanoparticles (AuNPs), which have strong surface plasmon resonance, Rayleigh scattering, (RRS) and a surface plasmon resonance (SPR) absorption (Abs) effect at 370 nm and 575 nm, respectively. Compled the new catalytic amplification indicator reaction with the specific As^3+^ aptamer reaction, a new RRS/Abs dual-mode aptamer sensor for the assay of trace As^3+^ was developed, based on the RRS/Abs signals increasing linearly with As^3+^ increasing in the ranges of 5–250 nmol/L and 50−250 nmol/L, whose detection limits were 0.8 nmol/L and 3.4 nmol/L As^3+^, respectively. This analytical method has the advantages of high selectivity, simplicity, and rapidity, and it has been successfully applied to the detection of practical samples.

## 1. Introduction

Carbon dots (CDs) are a new type of organic–inorganic hybrid carbon nanomaterial that have outstanding characteristics, such as strong fluorescence, good stability in aqueous solution, weak toxicity, many ways to obtain raw materials, low cost, easy preparation, biocompatibility, and biodegradability [1]. CDs were discovered unintentionally in the process of the purification of fluorescent single-walled carbon nanotube fragments by Xu et al. [2] in 2004. With the in-depth study of CDs, their synthesis and application have attracted the attention of researchers. At present, the main preparation methods for CDs are the top-down method and the bottom-up method. The top-down method is used to destroy large carbon clusters by chemical oxidation or physical cracking to prepare CDs, such as the electrochemical method. The bottom-up method uses different carbon-based precursors to prepare CDs through a series of chemical reactions. The main methods commonly used are hydrothermal synthesis, ultrasonic synthesis, and microwave-assisted synthesis [3]. It is a common strategy to use the fluorescence characteristics of CDs to construct analysis methods. Li et al. [4] used o-phenylenediamine (o-PD) and hydroquinone (HQ) as precursors, prepared the CDs with different surface states via oxidation/polymerization and a Schiff base reaction at room temperature, and applied them to fluorescent sensors. Zhao et al. [5] prepared the contamination of waste cellulose diacetate (CDA) from discarded cigarette filters as a precursor to prepare fluorescent N-doped carbon dots (N-CDs) via a one-pot hydrothermal carbonization in an aqueous solution with a low-cost ammonium hydroxide as the passivation agent, and they then applied it to the fluorescence-sensing detection of tetracycline (TC). In order to improve the selectivity and sensitivity of the method, researchers combined CDs with aptamers (Apts) to construct a series of sensors [6,7]. Apts not only have the specificity of antibodies but also the advantages of good thermal stability, easy synthesis, modification, production, storage, and wide application. Wang et al. [8] developed a new high-sensitivity fluorescent aptasensor for the detection of ochratoxin A (OTA) based on the tuning aggregation/disaggregation behavior (fluorescence quenching) of graphene quantum dots (GQDs) by structure-switching aptamers. An electrochemical biosensing strategy was developed for the green and ultrasensitive detection of tumor cells by combining aptamer–DNA, concatamer-CdTe quantum dots (QDs), and a signal amplification probe with mercury-free anodic stripping voltammetry (ASV) by Zheng et al. [9]. At present, there are a number of reports regarding the applicability of fluorescent CDs to the detection of metal ions in the environment and their many advantages. Wang et al. [10] prepared gold-doped carbon dots, using fullerene as a precursor to microwave irradiation, and successfully applied them to the catalytic amplification detection platform of As^3+^, realizing the sensitive detection of As^3+^. There is no report about the microwave preparation of CD nanosols by CA-EDA and its application to aptamer-mediated catalytic amplification–nanogold RRS/Abs analysis platforms.

As far as we know, the strong coupling between metal nanoparticles and light with specific photon energy is due to the optical excitation of metal collective electron resonances, which is called surface plasmon. Among them, AuNPs have strong surface plasmon effects, such as SPR absorption and RRS [11]. RRS is one of the most simple, rapid, and sensitive scattering spectral techniques. It is affected by the molecular structure, size, and shape of nanoparticles; charge distribution; and interface properties. It has been used in the detection of biomacromolecules, environmental pollutants, proteins, and so on [12,13,14]. Ephedrine (EH) and pseudoephedrine (PEH) could make different changes in the RRS spectrum of the detection system, which used Ce^3+^ functionalized gold nanoparticles as a probe [15]. Based on the different catalytic effects of tobramycin (TOB) aptamer-modified AuNPs and different concentrations of AuNPs-aptamers catalyzing the reduction reaction of Cu_2_O, Yan et al. constructed an RRS sensor for the detection of TOB and applied it to the detection of TOB in milk samples [16]. The nanoparticles’ absorption peak is produced by their SPR, which can be scanned and recorded by a spectrophotometer. Spectrophotometry has always been a research hotspot because of its simplicity, easy observation and operation, low cost, and other characteristics [17,18]. At present, a colorimetric sensor based on AuNPs has been reported. A detection strategy for colorimetric bacteria in water, based on 4-mercaptophenylboronic acid (4-MPBA) and functionalized gold nanoparticles (AuNPs), was introduced by Huang et al. [19] Citrate-capped gold nanoparticles as a sensing probe for the determination of cetyltrimethylammonium surfactants using FTIR spectroscopy and colorimetry [20]. The single spectrum method is a common analysis method, but it has some defects, such as stability, sensitivity, and accuracy. In order to improve this situation, researchers used the combination of bispectral to improve the defect of the analysis method and to make the method more sensitive, stable, and accurate [21,22]. To date, some bispectral methods have been reported to detect the same target. Fu et al. reported a fluorescent and colorimetric dual-mode nanosensor for the detection of tetracycline (TC) based on reduced state carbon dots (r-CDs) [23]. Han et al. realized dual-signal responsive optical sensors by combining RRS and the colorimetry detection of glutathione (GSH) based on the etching effect of GSH on MnO_2_ nanoflakes [24]. To date, there is no report about the RRS/Abs dual-mode sensing platform based on aptamer-regulated AuNPs and their application in the detection of trace As^3+^.

Arsenic ion (As^3+^) is a well-known toxic heavy metal element that widely exists in the environment. The frequent occurrence of arsenic pollution has posed a serious threat to human health and the environment [25]. In severe cases, it can lead to cancer or even death. In addition, an experiment also confirmed that As (III) has carcinogenic effects [26]. Therefore, with the increase in people’s attention to heavy metal pollution in the environment, it is particularly important to develop a sensitive, rapid, and convenient As^3+^ detection method. The methods that have been reported for the determination of arsenic ions include electrochemical biosensors (EBS) [27], colorimetry [28], atomic absorption spectrometry (AAS) [29], and liquid chromatography (LC) [30]. AAS and LC need pretreatment and expensive instruments, while EBS needs complicated operation steps and is time consuming. Colorimetry is simple, but its sensitivity is low. These methods are unable to perform the sensitive and rapid detection of As^3+^. In order to meet the needs of As^3+^ detection in water, a CD_N_ with high catalytic activity and stability was prepared by the CA-EDA microwave method, and its nanocatalytic indicator reaction was combined with the aptamer reaction to construct the RRS/Abs dual-mode sensing platform for the detection of As^3+^ in this paper.

## 2. Materials and Methods

### 2.1. Materials

An F-7000 fluorescence spectrophotometer (Hitachi Co., Tokyo, Japan); a TU-1901 dual-beam UV−visible spectrophotometer (Beijing Puxi General Equipment Limited Co., Beijing, China); an HH-S2 digital display thermostatic water bath (Changzhou Guohua Electric Appliance Co., Ltd. Changzhou, China); a KQ3200DB numerical control ultrasonic cleaner (Kunshan Ultrasonic Instrument Co., Ltd., Kunshan, China); a JUPITER-B microwave digestion instrument (Shanghai Xinyi Microwave Chemical Technology Co., Ltd., Shanghai, China); and a UPW-N series water purifier (Shanghai Yidian Scientific Instrument Co., Ltd., Shanghai, China) were used.

A stock solution of 100 μmol/L of Apt–As was prepared with a sequence of 5′-ATG CAA ACC CTT AAG AAA GTG GTC CAA AAA ACC ATT G-3′ and diluted to the required concentration when used. A solution of 1 mol/L of citric acid (CA) was prepared as follows: 21 g of citric acid (monohydrate) was diluted in a 100 mL volumetric flask with water. An amount of 1 mol/L of ethylenediamine solution was prepared as follows: 6.667 mL of ethylenediamine solution was diluted in a 100 mL volumetric flask with water. We took anhydrous ethanol and distilled water (V/V, 1:1) to obtain a 49.85% ethanol solution. A total of 1.0 mg/mL of HAuCl_4_ (Shanghai McLean Biochemical Co., Ltd., Shanghai, China) was prepared as follows: took 250 μL 4% HAuCl_4_ into a centrifuge tube and made up to 10 mL with water. We used a 10 μmol/L As^3+^ solution and 0.01 mol/L HCl (Sichuan Xilong Science Co., Ltd., Chengdu, China). All reagents were of analytical grade, and the water was double distilled.

### 2.2. Preparation of CD_N_

The amounts of 1 mL of 1 mol/L CA and 3 mL of 1 mol/L EDA were evenly mixed, and the volume was adjusted to 25 mL. Then, the solution was transferred to a microwave digestion apparatus with a microwave power of 500 W, 200 °C and heated for 60 min. After the reaction was completed, it was cooled to room temperature and then centrifuged for 10 min at 10,000 r/min. Finally, a concentration of 8.4 mg/mL CD_N_ was obtained (calculated as CA).

### 2.3. Experimental Methods

In a 5.0 mL stoppered tube, 70 μL of 84 μg/mL CD_N_ solution, 80 μL of 1 μmol/L Apt_As_ solution, and a certain concentration of As^3+^ solution were added successively, mixed well, and left for 15 min. The amounts of 400 μL of 49.85% Et, 100 μL of 0.01 mol/L HCl, and 140 μL of 1 mg/mL HAuCl_4_ were added in order to make the volume of 2 mL, mixed well, reacted in an 85 °C water bath for 20 min, and then the temperature was rapidly cooled with ice water. Under the conditions of 350 V and excitation of slit = emission (slit = 5 nm), the RRS spectrum was obtained using a fluorescence spectrophotometer. The RRS peak intensity I at 370 nm was measured. As^3+^-free was a blank value of I_0_, calculated as ∆I = I − I_0_. Furthermore, the Abs spectrum of the system was obtained by dual-beam UV spectrophotometer scanning, and the Abs intensity at 575 nm was determined as A. Similarly, As^3+^-free was a blank value of A_0_, calculated as ΔA = A − A_0_.

## 3. Results and Discussion

### 3.1. Analytical Principle

**Figure 1:**
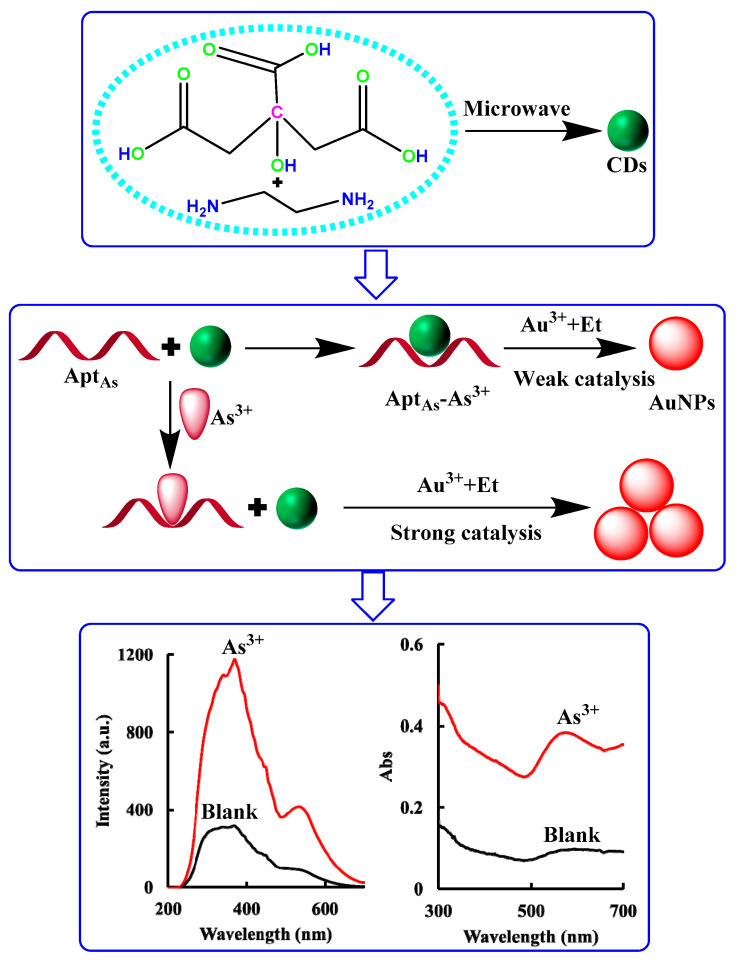
Principle of aptamer mediated nitrogen-doped carbon dot (CD_N_) catalytic amplification–AuNP RRS/Abs dual-mode detection of trace As^3+^.

The reaction of Et–HAuCl_4_ was slow without a catalyst in the system, less AuNPs were generated, and the RRS scattering signal and Abs absorption value were weak. The CD_N_ had a strong catalytic effect on the Et–HAuCl_4_ reaction, which was able to generate more AuNPs and enhance the RRS/Abs signal of the system. After adding Apt_As_, it bound to the surface of the CD_N_ through an electrostatic interaction, and the CD_N_ was encapsulated and inhibited its catalytic activity. When the analyte As^3+^ was added, it specifically combined with Apt_As_ to form a stable structure and fell off the surface of the catalytyst CD_N_. Then, the catalytic performance was enhanced by increasing the As^3+^ content, and the amount of AuNPs was gradually increased. The RRS signal of the system at 370 nm was increased gradually, and the Abs signal value at 575 nm was also enhanced gradually. Based on this, a sensitive and simple RRS/Abs dual-mode analysis method for the determination of trace As^3+^ was established (Figure 1).

### 3.2. Characterization of CD_N_

#### 3.2.1. X-ray Diffraction (XRD) and Infrared Spectrum (IR)

**Figure 2:**
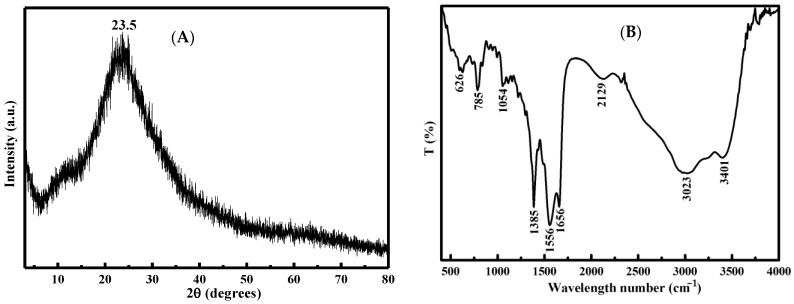
(**A**): XRD of CD_N_; (**B**): IR of CD_N_.

From the XRD pattern of the CD_N_, it can be seen that the CD_N_ is at 23.5° (Figure 2A). The results indicate that the CD_N_ had an amorphous and disordered structure [31]. It can be seen from the infrared spectrum that the CD_N_ had absorption at 3401 cm^−1^, 3023 cm^−1^, 2129 cm^−1^, 1656 cm^−1^, 1556 cm^−1^, 1385 cm^−1^, 1054 cm^−1^, 785 cm^−1^, and 626 cm^−1^. Among them, the absorption peaks at 3401 cm^−1^, 3023 cm^−1^, 1656 cm^−1^, 1385 cm^−1^, and 1054 cm^−1^ belong to the stretching vibrations of -O-H, -C-H, -C = O, -C-N, and -C = C-, respectively. The peak at 2129 cm^−1^ is the sum frequency absorption band vibration of H_2_O, the peak at 1556 cm^−1^ belongs to in-plane bending vibration of -N-H, the peak at 785 cm^−1^ belongs to torsional deformation of -NH_2_, and the peak at 626 cm^−1^ belongs to out-of-plane bending vibration of -C-OH (Figure 2B).

#### 3.2.2. Transmission Electron Microscopy (TEM) and Energy Dispersive Spectroscopy (EDS)

**Figure 3:**
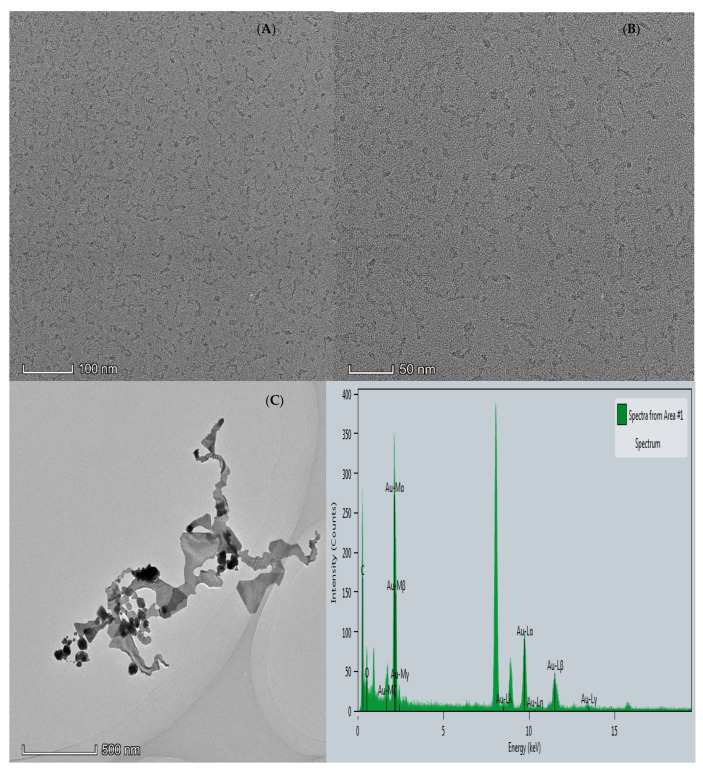

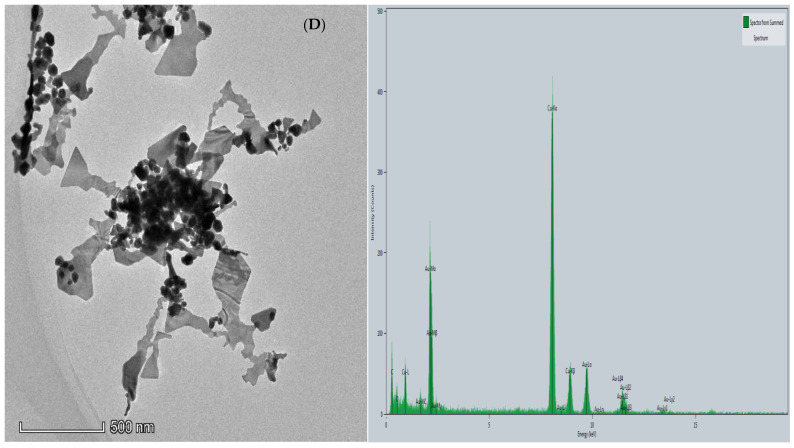
(**A**,**B**): TEM of CD_N_; (**C**): 40 nmol/L Apt_As_ + 2.94 μg/mL CD_N_ + 9.97% Et + 0.5 mmol/L HCl + 0.07 mg/mL HAuCl_4_; (**D**): C + 150 nmol/L As^3+^.

After the CD_N_ and AuNP sample solution was prepared according to the experimental method, the morphology of the samples was recorded with a transmission electron microscope. It can be seen from Figure 3A,B that the CD_N_ has spherical nanoparticles with a particle size of about 8 nm. It can be seen from Figure 3C that the system produced less AuNPs in the presence of an Apt, and the gold nanoparticles were spherical and flaky structures. With the addition of As^3+^, it combined with the Apt to form a stable structure and desorbed from the surface of the CD_N_, and the catalytic activity of the CD_N_ was restored, resulting in the increase in AuNPs and the formation of gold nanoparticles with spherical and flaky structures (Figure 3D). From the energy spectrum of Figure 3C,D, it can be observed that gold nanoparticles are formed in the system.

#### 3.2.3. Fluorescence, RRS, and Abs Spectra of CD_N_

**Figure 4:**
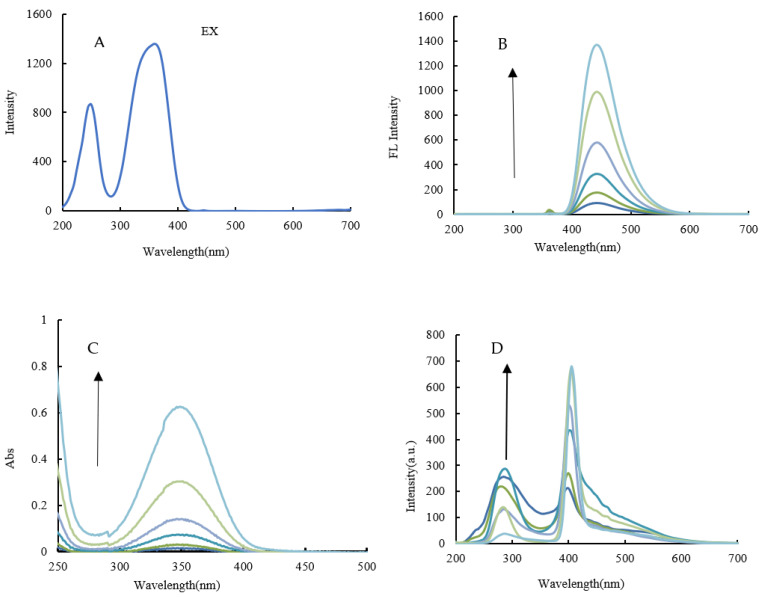
Excitation (**A**), fluorescence (**B**), Abs (**C**), and RRS (**D**) spectra of CD_N_. A: excitation spectra of CD_N_; B: emission spectra of CD_N_, (2.625, 5.25, 10.5, 21, 42, 84) μg/mL CD_N_; C: Abs spectra of CD_N_, (26.25, 52.5, 105, 210, 420, 840) μg/mL CD_N_; D: RRS spectra of CD_N_, (2.625, 5.25, 10.5, 21, 42, 84) μg/mL CD_N_.

With a voltage of 350 V and an excitation slit = emission slit of 5 nm, as well as an excitation wavelength of 250 nm, the CD_N_ has a fluorescence peak at 442 nm. Then, the optimal excitation wavelength EX = 362 nm is determined under the condition of EM = 442 nm (Figure 4A). As the increase of CD_N_ concentration the intensity of the fluorescence peak at 442 nm gradually increases (Figure 4B). The CD_N_ has a UV absorption peak at 350 nm, and the absorption peak at 350 nm gradually increases with the increase in the CD_N_ concentration (Figure 4C). Similarly, the CD_N_ also has an RRS peak at 400 nm. As the CD_N_ concentration increases, the intensity at 400 nm gradually increases (Figure 4D).

### 3.3. RRS and Abs Spectra of CD_N_ Catalytic Amplification System

**Figure 5:**
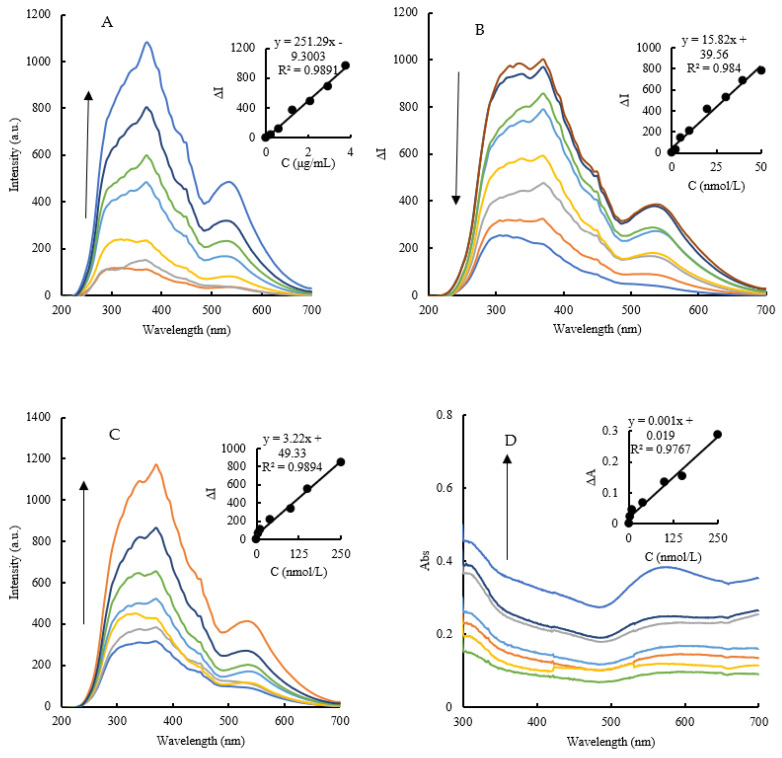
RRS and Abs spectra of the CD_N_ catalytic amplification system. (**A**): RRS spectra of CD_N_–Et–HAuCl_4_ system, (0, 0.21, 0.63, 1.26, 2.1, 2.94, 3.78) μg/mL CD_N_ + 9.97% Et + 0.5 mmol/L HCl + 0.07 mg/mL HAuCl_4_; (**B**): RRS spectra of Apt_As_–CD_N_–Et–HAuCl_4_ system, (0, 2.5, 5, 10, 20, 30, 40, 50) nmol/L Apt_As_ + 2.94 μg/mL CD_N_ + 9.97% Et + 0.5 mmol/L HCl + 0.07 mg/mL HAuCl_4_; (**C**): RRS spectra of As^3+^–Apt_As_–CD_N_–Et–HAuCl_4_ system, (0, 5, 10, 40, 100, 150, 250) nmol/L As^3+^ + 40 nmol/L Apt_As_ + 2.94 μg/mL CD_N_ + 9.97% Et + 0.5 mmol/L HCl + 0.07 mg/mL HAuCl_4_; (**D**): Abs spectra of As^3+^–Apt_As_–CD_N_–Et–HAuCl_4_ system, (0, 5, 10, 40, 100, 150, 250) nmol/L As^3+^ + 40 nmol/L Apt_As_ + 2.94 μg/mL CD_N_ + 9.97% Et + 0.5 mmol/L HCl + 0.07 mg/mL HAuCl_4_.

Under the experimental conditions, the Et–HAuCl_4_ system slowly forms AuNPs, and the system generates less AuNPs. After adding a certain amount of the CD_N_, it has a significant catalytic effect on the reaction to form AuNPs. The generated AuNPs have two RRS peaks at 370 nm and 535 nm, and the RRS peak at 370 nm changes obviously (Figure 5A). After the Apt is added to the system, it binds to the surface of the CD_N_ and inhibits its catalytic activity through electrostatic action, resulting in less AuNPs and the system RRS signal decreases (Figure 5B). When the analyte As^3+^ is added to the inhibition system, Apt_As_ can specifically bind with As^3+^ to form a stable structure, which will fall off from the surface of the CD_N_. Furthermore, the catalytic ability of the CD_N_ catalyst is restored, resulting in an increase in AuNPs and the recovery the of RRS signal. As shown in Figure 5C, within a certain range, as the concentration of As^3+^ increases, the intensity of the system RRS signal gradually increases, so a new RRS method for As^3+^ detection is established. Within a certain concentration range, as the concentration of As^3+^ is increasing in the As^3+^–Apt_As_–CD_N_–Et–HAuCl_4_ system, the color changes from lavender to dark purple, which is attributed to the increase in the AuNPs generated by the system (Figure 5D). The Abs signal of the system increases gradually, and an absorption peak is produced at 575 nm, which belongs to the SPR absorption peak of AuNPs. The RRS assay was more sensitive than the Abs, but the cost of the Abs assay was lower than that of the RRS. Thus, the two assays have their own advantages.

### 3.4. Optimal Analytical Conditions

**Figure 6:**
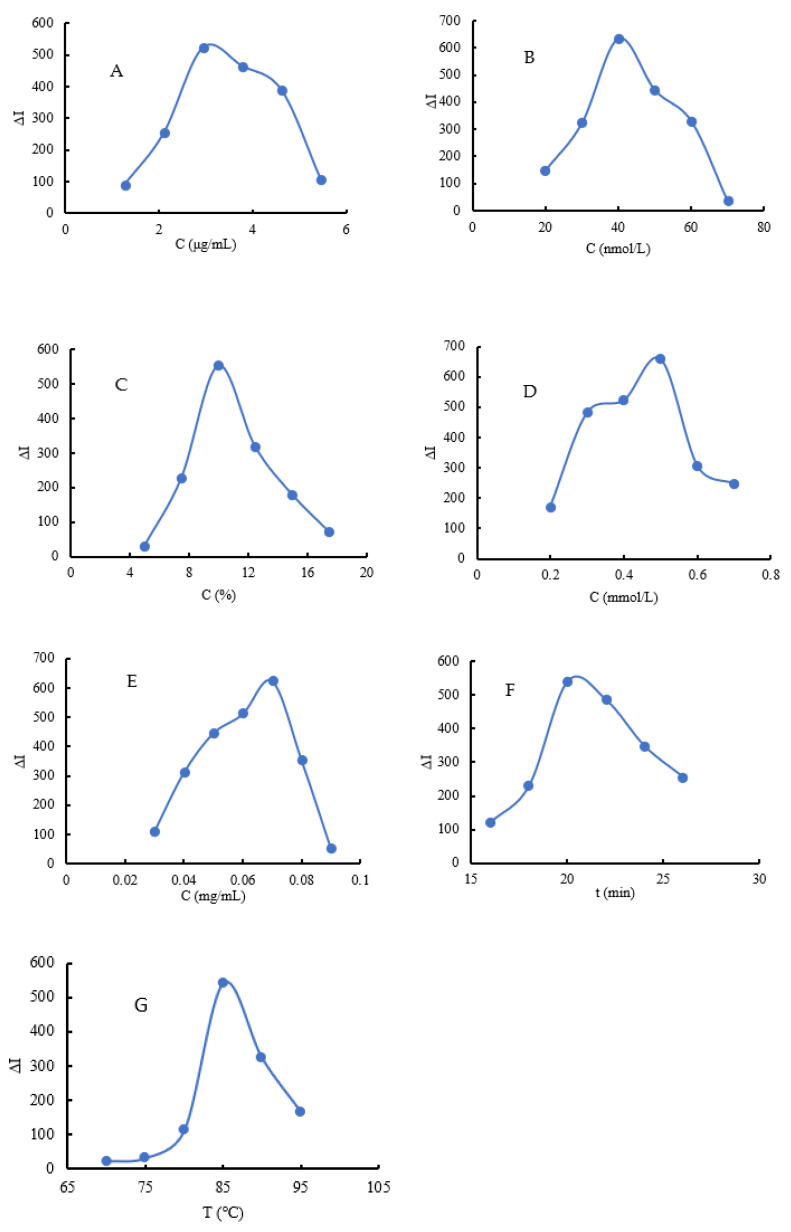
Optimization of analytical conditions. (**A**): the concentration of CD_N_; (**B**): the concentration of Apt_As_; (**C**): the percentage of Et; (**D**): the concentration of HCI; (**E**): the concentration of HAuCl_4_; (**F**): the reaction time; and (**G**): the reaction temperature.

We studied the reaction conditions of the As^3+^–Apt_As_–CD_N_–Et–HAuCl_4_ system according to the experimental methods. The results show that the CD_N_ concentration is 2.94 μg/mL (Figure 6A), the Apt_As_ concentration is 40 nmol/L (Figure 6B), the percentage of Et is 9.97% (Figure 6C), the HCl concentration is 0.5 mmol/L (Figure 6D), the HAuCl_4_ concentration is 0.07 mg/mL (Figure 6E), the reaction time is 20 min (Figure 6F), the reaction temperature is 85 °C (Figure 6G), and the ∆I value of the system reaches the maximum. Therefore, these conditions were chosen as experimental conditions.

### 3.5. Influence of Coexisting Substances

Based on the experimental method, the effect of coexisting substances on As^3+^ detection was investigated. The results show that when the As^3+^ concentration is 150 nmol/L, the relative error is within ± 10% and common amino acids and ions have no effect on the measurement of As^3+^ (Table 1), which indicates that the method has a good selectivity. When the Fe (III) concentration is greater than 4.5 μmol/L, that is, 30 times that of As^3+^, it is light yellow and hydrolysis, which both affect the RRS signal that will interfere with the detection of As^3+^. Similar to Fe (III), when the Cr (III) concentration is greater than 15 μmol/L, it will also interfere with the detection of As^3+^ due to the light blue color and its hydrolysis. The NO^2−^ can be oxidized by Au (III), and when it is greater than 7.5 μmol/L, it will also interfere with the detection.

### 3.6. Working Curves

Under the optimal experimental conditions, according to the concentration of As^3+^ and RRS/Abs signal changes of the system, the relationship between the As^3+^ concentration and the RRS/Abs signal was good and linear. In the RRS detection method, the linear range of As^3+^ concentration is 5−250 nmol/L, the linear equation between As^3+^ concentration and RRS intensity ∆I_370 nm_ is ∆I_370 nm_ = 3.22C + 49.33, and the correlation coefficient is 0.9894. In the Abs detection method, the linear range of As^3+^ concentration is 50−250 nmol/L, the linear equation between As^3+^ concentration and Abs intensity ∆A_575 nm_ is ∆A_575 nm_ = 0.001C + 0.019, and R^2^ is 0.9767. The standard deviation (Sd = 3.39) is obtained by the parallel measurement of blank samples 11 times, and the formula LOD = 3 × Sd/N (N is the slope of a working curve) can calculate the detection limits of the two methods that are 0.8 nmol/l and 3.4 nmol/l, respectively. Compared with the reported analytical methods for the determination of As^3+^, this method is simple, sensitive, and rapid (Table 2).

### 3.7. Analytical Application

The sample pretreatment was as follows: 20 mL of industrial wastewater (A, B, and C) was taken, filtered to remove the suspended particles, and centrifuged at 10,000 r/min for 10 min; then, the supernatant was obtained and used as the actual test sample. According to the experimental method, the samples were detected, and the obtained results are shown in Table 3. The recovery is between 98.67–108.67%, and the relative standard deviation (RSD) is between 1.89–2.84%.

## 4. Discussion

In this paper, a stable nitrogen-doped CD nanosol with high catalytic activity was prepared using the microwave digestion method and characterized by electron microscopy and molecular spectroscopy. It had been found that the CD_N_ had a strong catalytic effect on the Et–HAuCl_4_ system to generate AuNPs, and the generated AuNPs had a strong RRS signal at 370 nm and an Abs absorption peak at 575 nm. After adding Apt_As_, Apt_As_ could combine with the CD_N_ and inhibit its catalysis as well as reducing the RRS/Abs signal of the system. When As^3+^ was added to the solution, the As^3+^ combined with the Apt_As_ to form a stable complex, releasing the CD_N_ and restoring its catalytic capabilities, so the RRS and Abs signals of the system were restored. According to this principle, a new RRS/Abs dual-mode sensing platform was established for the determination of As^3+^. Through the determination and analysis of actual industrial wastewater samples, this analytical method had the characteristics of high selectivity, simplicity, and rapidity, and it could meet the needs of the detection of arsenic ions in water. Compared with the reported analytical methods for the determination of As^3+^, Li et al. [32] proposed the detection of arsenic based on MIL–AuNPs/PSi having excellent sensitivity and stability in the detection of probe molecules. This method had high sensitivity, but the operation was complex. Gu et al. [33] used the electrochemical biosensor detection of As^3+^, which had highly sensitive good selectivity, but the process was complex. The fluorescence aptamer sensor detection of As (III) was established by Taghdisi et al. [34], the method had good selectivity but its operation was complicated. Zhang et al. [21] proposed aptamer-mediated N/Ce-doped carbon dots as an RRS/FL dual-mode probe for arsenic (III), and the characteristics of this method were good selectivity and a simple but narrow linear range. Yang et al. [35] established the colorimetric detection of arsenic (III) based on the peptideinduced aggregation of gold nanoparticles through screening arsenic (III)-binding peptides with traits of simplicity and low sensitivity. Ma et al. [36] proposed a new electrochemical sensor based on ion imprinted polymer (IIP)–nanoporous gold (NPG)-modified gold electrodes (IIP/NPG/GE) for the determination of As^3+^. This method was highly selective and ultrasensitive for the detection of As^3+^, but the time-consuming nature of the incubation steps in the sensor detection process affected the detection time of samples. Therefore, our method has good selectivity and easy operation, in addition to being simple, sensitive, and rapid (Table 2). Our research will provide the basis and theoretical guidance for the detection of other toxic heavy metals, which is of great significance to environmental protection and quality. Further work will be to apply this method to the detection of other toxic and harmful heavy metal irons. In conclusion, the developed dual-mode sensor platform may be a very promising and widely used analytical tool.

## Figures and Tables

**Table 1:** Influence of coexisting substances.

Coexisting Substance	Tolerance Limit (Times)	Relative Error (%)	Coexisting Substance	Tolerance Limit (Times)	Relative Error (%)
NH_4_^+^	100	−1.3	Ca^2+^	100	−3.2
Mg^2+^	100	−2.6	K^+^	100	6.5
Ba^2+^	100	−5.2	Cu^2+^	100	3.3
Zn^2+^	100	3.6	NH_4_^+^	100	8.2
IO_3_^−^	100	−7.6	Fe^3+^	30	3.2
Na^+^	100	8.3	Cr^6+^	80	6.3
Mn^2+^	100	4.7	Cr^3+^	100	4.9
Al^3+^	100	3.1	NO_2_^−^	50	2.3
I^−^	100	8.2	SO_4_^2−^	100	3.6
Pb^2+^	100	−6.2	PO_4_^3−^	100	−1.4

**Table 2:** Comparison of some reported spectral methods for As^3+^.

Method	Principle	Linear Range	Detection Limit	References
SERS	Magnetic ionic liquid (MIL)–gold nanoparticles/porous silicon composite substrates were synthesized using an MIL-assisted strategy and successfully applied to the detection of arsenic.	2–80, 200–800 ppb	0.5 ppb	[32]
Electrochemical biosensor	Based on the gold electrode assembled by DNA and the signal amplification strategy of hybridization chain reaction (HCR) and RecJf exonuclease.	0.1–200 ppb	0.02 ppb	[33]
Fluorescence aptamer sensor	Based on the complementary chains of Streptomyces silica nanoparticles with a free and Fas-labeled aptamer.	2–500 nmol/L	0.45 nmol/L	[34]
RRS/FL dual-mode	Base on Apt-mediated N/Ce-doped carbon dots, the specific reaction of aptamer–As (III), and RRS/FL dual-mode probe detection of As (III).	0.5–5.8 μg/L	0.2 μg/L	[21]
Colorimetry	Based on As (III)-binding, peptide-modified AuNPs, and the competitive binding of As (III)–As (III)-binding peptides.	195–600 nmol/L	54 nmol/L	[35]
Electrochemical sensor	Based on ion-imprinted polymer (IIP) and nanoporous gold-modified gold electrode, an electrochemical sensor for the determination of As^3+^.	2.0 × 10^−3^–9.0 nmol/L	7.1 × 10^−3^ nmol/L	[36]
RRS/Abs	Coupled CD catalytic reaction and the specific reaction of Apt_As_–As^3+^. RRS/Abs increased with an increase in As^3+^.	5–250 nmol/L 50–250 nmol/L	0.8 nmol/L 3.4 nmol/L	This method

**Table 3:** Analytical results of As^3+^ in samples.

Sample	Single Measured Value (nmol/L)	Average Value (nmol/L)	Added As^3+^ (nmol/L)	Found As^3+^ (nmol/L)	Recovery (%)	RSD (%)	Content (μmol/L)
A	153, 145, 149, 155, 150	150.4 ± 3.85	30	180	98.67	2.56	3.008
B	180, 176, 185, 182, 183	181.2 ± 3.42	30	212	102.67	1.89	5.436
C	175, 187, 179, 185, 186	182.4 ± 5.18	30	215	108.67	2.84	5.472

## Data Availability

The raw/processed data required to reproduce these findings cannot be shared at this time as the data also forms part of an ongoing study.

## Sample Availability

Not available.
